# Associations between VDR gene polymorphisms and colorectal cancer susceptibility: an updated meta-analysis based on 39 case-control studies

**DOI:** 10.18632/oncotarget.23964

**Published:** 2018-01-04

**Authors:** Zhipeng Pan, Mengya Chen, Xingxing Hu, Hua Wang, Jiajia Yang, Congjun Zhang, Faming Pan, Guoping Sun

**Affiliations:** ^1^ Department of Medical Oncology, The First Affiliated Hospital of Anhui Medical University, Hefei, Anhui, 230032, China; ^2^ Department of Epidemiology and Biostatistics, School of Public Health, Anhui Medical University, Hefei, Anhui, 230032, China; ^3^ The Key Laboratory of Major Autoimmune Diseases, Anhui Medical University, Hefei, Anhui, 230032, China

**Keywords:** vitamin D receptor, colorectal cancer, VDR, meta-analysis, polymorphisms

## Abstract

**Background:**

Recent studies have reported the associations between vitamin D receptor (*VDR*) polymorphisms and colorectal cancer (CRC), but the results were not always consistent. This meta-analysis aims to evaluate whether *VDR* polymorphisms are associated with CRC susceptibility.

**Materials And Methods:**

Studies on the associations between *VDR* polymorphisms and CRC were retrieved from the Web of Science, PubMed, the Chinese Biomedical Database (CBM), Chinese National Knowledge Infrastructure (CNKI) and Wanfang (Chinese) databases. The odds ratio (OR) with 95% confidence intervals (*CIs*) was obtained.

**Results:**

Thirty-nine articles met all inclusion criteria and were included in the meta-analysis including 22101 CRC cases and 23696 healthy controls. The 39 articles consisted of five *VDR* gene polymorphisms including *ApaI, FokI, BsmI, TaqI* and *Cdx2*. The results of meta-analysis showed that the *FokI* polymorphism was on the fringe of statistically significant in the comparisons of F allele vs. f allele in fixed model (OR = 1.029, 95% CI = 0.999–1.059, P_raw_ = 0.057, P_FDR_ = 0.057). Moreover, for the associations between *BsmI* polymorphism with CRC, We observed significant differences in allele frequencies, the homozygous model and the dominant model between CRC patients and healthy controls (B vs. b: OR = 0.862, 95% CI = 0.761–0.976, P_raw_ = 0.019, P_FDR_ = 0.019; BB vs. bb: OR = 0.786, 95% CI = 0.636–0.972, Praw = 0.026, P_FDR_ = 0.039; BB + Bb vs. bb: OR = 0.934, 95% CI = 0.888-0.982, Praw = 0.008, P_FDR_ = 0.024, respectively).

**Conclusions:**

This meta-analysis suggests that *BsmI* is associated with CRC risk and *FokI* might be a risk factor for CRC. However, these associations with CRC need further studied.

## INTRODUCTION

Colorectal cancer (CRC) is a major health problem and is currently ranked third for both cancer incidence and mortality [1]. In spite of the revised treatment patterns, CRC remains a major cause of cancer mortality. At an estimated 1.2 million new cancer cases and 608,700 deaths worldwide each year, people who die from CRC account for 8% of all cancer related deaths [2]. The incidence rate of CRC has been increasing greatly in China in the past few years, which accounts for about 6.5% of total cancers in urban areas and 4.6% in rural areas [3]. CRC is a multifactorial disease, involving the complex interactions between environmental and genetic factors [4]. However, the exact mechanisms which result in the development of colorectal cancer remain unclear. Nowadays, a large number of candidate genes responsible for the genesis of colorectal cancer have been identified.

Recently, the associations between vitamin D and colorectal cancer has aroused a great deal of attention, and genetic variation in metabolic pathways for these nutrients may play a role in colorectal carcinogenesis [5]. It’s known to us that Vitamin D plays an important role in calcium absorption, cellular proliferation and differentiation, as well as carcinogenesis. Animal studies and case–control studies in humans have provided strong evidence that vitamin D protects against colorectal cancer [6, 7]. Genomic actions of the active metabolite of vitamin D [1, 25(OH)_2_D_3_] are mediated by the vitamin D receptor (VDR) which maps to a region on chromosome 12 [8, 9]. The active form of vitamin D [1,25(OH)_2_D_3_] is bound by the intracellular *VDR*. This complex bindings and interactions with target-cell nuclei (at *VDR* elements) produce varieties of biological effects [10]. Recently, the *VDR* gene polymorphisms [11–49] including *FokI* [12, 13, 15, 18, 19, 21–26, 29–32, 35–39, 41–43, 45–47, 49], *BsmI* [11–13, 15–18, 20, 23, 26, 27, 29, 30, 32, 33, 36, 37, 40–42, 44, 48, 49], *ApaI* [11–13, 15, 16, 18, 23, 27, 28, 30, 36, 41], *TaqI* [12–15, 17, 18, 20–24, 27, 28, 31, 36, 38, 41, 49] and *Cdx2* [21, 30, 31, 36] have been assessed in genetic associations studies of CRC, but the results from these studies are still inconsistent. Three meta-analyses [3, 50, 51] had been published assessing the associations between *VDR* polymorphisms and CRC risk in recent years. However, there are some limitations in the three studies, such as relatively small sample size. Moreover, a number of studies that assessed the associations between *VDR* polymorphisms and CRC risk were published after that period. In order to derive a more comprehensive estimation of the associations between *VDR* polymorphisms and CRC risk, we conducted a meta-analysis from 39 eligible case-control studies to evaluate the associations.

## RESULTS

### Data source

**Figure 1:**
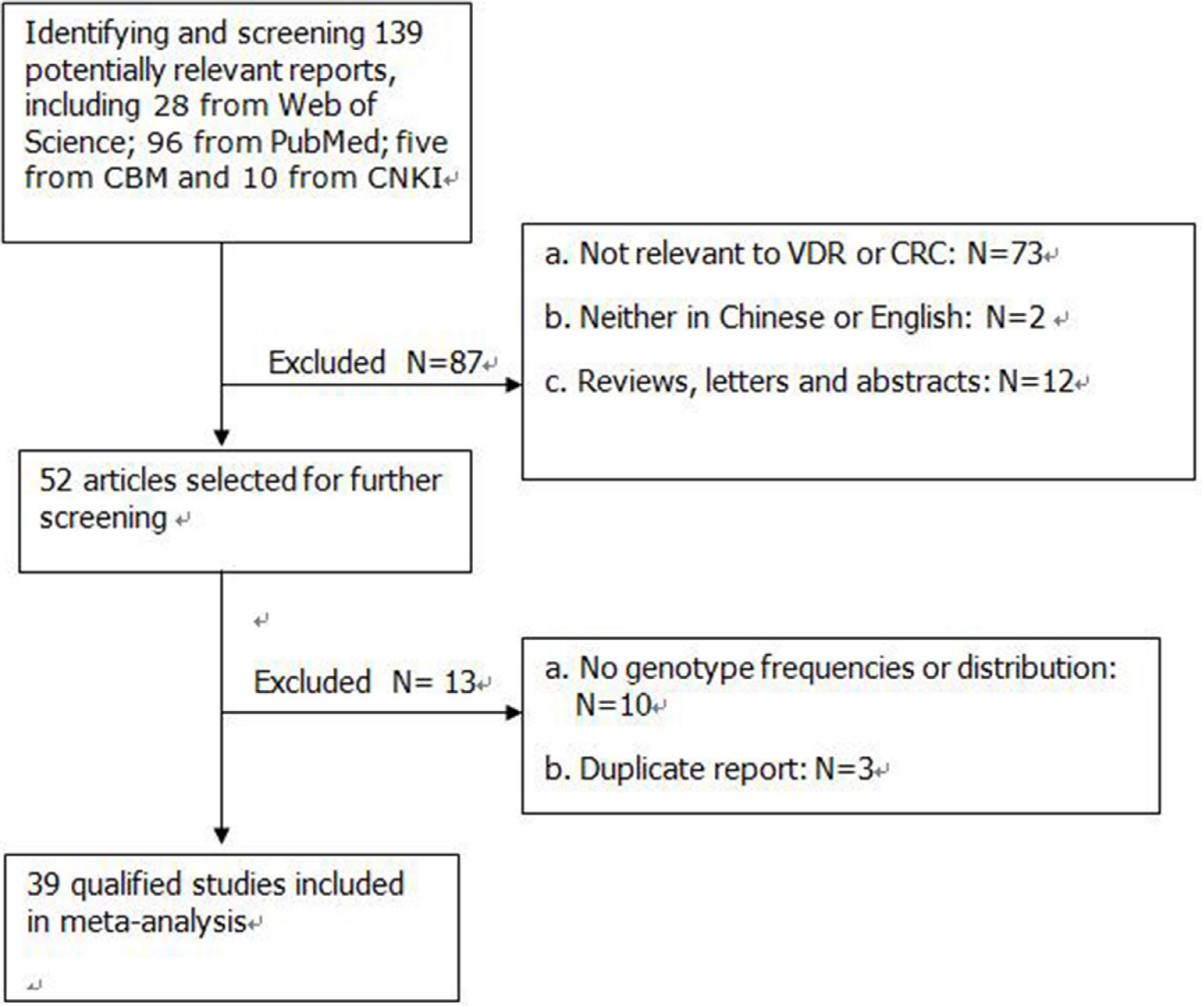
Flow diagram of the study selection process

Figure 1 summarizes the selection process of study. According to the strategy, 139 published studies relevant to the *VDR* genes and the risk of CRC were reviewed including 28 from The Web of Science; 96 from PubMed; five from CBM and 10 from CNKI. 52 articles were selected for full-text review on the basis of their titles and abstracts. Finally, 39 articles met all inclusion criteria and were included in the meta-analysis including 22101 CRC cases and 23696 healthy controls. The 39 articles [11–49] consisted of five *VDR* gene polymorphisms including *FokI* [12, 13, 15, 18, 19, 21–26, 29–32, 34–39, 41–43, 45–47, 49], *BsmI* [11–13, 15–18, 20, 23, 26, 27, 29, 30, 32, 33, 36, 37, 40–42, 44, 48, 49], *ApaI* [11–13, 15, 16, 18, 23, 27, 28, 30, 36, 41], *TaqI* [12–15, 17, 18, 20–24, 27, 28, 31, 36, 38, 41, 49] and *Cdx2* [21, 30, 31, 36]. Selected characteristics on the relationships between *VDR* polymorphisms and CRC were listed in Table 1.

**Table 1:** Characteristics of individual studies included in meta-analysis

First Author	Year	Country	Ethnicity	Case/ Control	age		Control Methods	HWE	VDR polymorphisms
				*N*	case	control			
Vigidal [11]	2016	Brazil	Caucasian	152/321	62.8 ± 13.02	62.7 ± 10.42	PCR-RFLP	Yes	BsmI, AapI
Alkhayal [12]	2016	Saudi Arabia	Caucasian	100/100	57.5 (20–80)	57.5 (21–81)	PCR	No	FokI, BsmI, AapI, TaqI
Takeshige [13]	2015	Japan	Asian	685/778	60.2 ± 9.1	58.6 ± 10.7	PCR-RFLP	Yes	FokI, BsmI, AapI, TaqI
Atoum [14]	2014	Jordan	Asian	93/102	NA	NA	PCR	Yes	TaqI
Laczmanska [15]	2014	Poland	Caucasian	179/180	65.7 (32–87)	NA	PCR	No	FokI, BsmI, AapI, TaqI
Rasool [16]	2014	India	Asian	180/188	52.05	51.06	PCR-RFLP	No	BsmI, AapI
Pibiri [17]	2014	United States	African American	961/838	62.0 ± 10	65.0 ± 6	PCR	Yes	BsmI, TaqI
Sarkissyan [18]	2014	American	Mixed	78/230	55.2 ± 9.9	54.9 ± 9.8	PCR-RFLP	Yes	FokI, BsmI, AapI, TaqI
Rasool [19]	2013	India	Asian	312/305	52.05	51.06	PCR	Yes	FokI
Gunduz [20]	2012	Turkey	Caucasian	43/42	54.8	48.8	PCR-RFLP	No	BsmI, TaqI
Bentley [21]	2102	New Zealand	Asian	200/200	69.5 ± 0.4	69.5 ± 0.4	Taqman	Yes	FokI, TaqI, Cdx2
Yamaji [22]	2012	Japan	Asian	684/641	NA	NA	Taqman	Yes	FokI, TaqI
Kupfer [23]	2011	United States	Mixed	2119/1975	64.5 ± 11.7	62.3 ± 13.2	Taqman	Yes	FokI, BsmI, AapI, TaqI
Ashktorab [24]	2011	United States	Caucasian	93/187	59	60	PCR	Yes	FokI, TaqI
Abulí [25]	2011	Spain	Caucasian	515/515	NA	NA	Taqman	Yes	FokI
Mahmoudi [26]	2010	Iran	Asian	452/452	44.3 ± 17.2	53.7 ± 13.3	PCR-RFLP	Yes	FokI, BsmI
Hughes [27]	2010	Czech Republic	Caucasian	754/627	61 (27–85)	53 (29–91)	ASM-PCR	Yes	BsmI, AapI, TaqI
Mahmoudi [28]	2010	Iran	Asian	160/180	52.6 ± 14.0	44.4 ± 17.6	PCR-RFLP	No	AapI, TaqI
Jenab [29]	2009	United Kingdom	Caucasian	1248/1248	58.5 ± 7.2	58.6 ± 7.2	Taqman	Yes	FokI, BsmI
Theodoratou [30]	2008	United Kingdom	Caucasian	3005/3072	62.0 ± 10.7	62.4 ± 10.5	Microarray	No	FokI, BsmI, AapI, Cdx2
Ochs-Balcom [31]	2008	United States	Mixed	250/246	62.7 ± 10.2	58.4 ± 12.1	Taqman	Yes	FokI, TaqI, Cdx2
Li [32]	2008	China	Asian	200/200	61.5 ± 12.6	61.3 ± 12.5	PCR-RFLP	Yes	FokI, BsmI
Parisi [33]	2008	Spain	Caucasian	50/32	NA	NA	PCR-RFLP	Yes	BsmI
Wang [34]	2008	China	Asian	60/218	38–78	19.6 ± 1.3	PCR-RFLP	Yes	FokI
Grünhage [35]	2008	Germany	Caucasian	194/220	65 ± 9	63 ± 8	PCR-RFLP	Yes	FokI
Flügge [36]	2007	Germany	Caucasian	256/256	61.9 ± 10.0	62.2 ± 11.2	PCR-RFLP	Yes	FokI, BsmI, AapI, TaqI, Cdx2
Slattery [37]	2007	United States	Caucasian	2380/2990	NA	NA	Taqman	Yes	FokI, BsmI
Yaylim-Eraltan [38]	2007	Turkey	Caucasian	26/52	59.1 ± 4.0	52.0 ± 0.8	PCR-RFLP	No	FokI, TaqI
Murtaugh [39]	2006	United States	Caucasian	1820/2821	NA	NA	PCR-RFLP	Yes	FokI
Kadiyska [40]	2006	Bulgaria	Caucasian	140/94	59 (22–83)	NA	PCR-RFLP	Yes	BsmI
Park [41]	2006	South Korea	Asian	190/318	55 (32–81)	NA	PCR-RFLP	Yes	FokI, BsmI, AapI, TaqI
Slattery [42]	2004	United States	Caucasian	1936/2130	NA	NA	PCR-RFLP	No	FokI, BsmI
Peters [43]	2004	United States	Caucasian	763/774	62.9	62.3	PCR-RFLP	Yes	FokI
Boyapati [44]	2003	United States	Caucasian	177/228	58.4 ± 8.4	56.0 ± 10.0	PCR-RFLP	No	BsmI
Wong [45]	2003	China	Asian	217/890	56.5	NA	PCR-RFLP	Yes	FokI
Peters [46]	2001	United States	Caucasian	239/228	NA	NA	PCR-RFLP	Yes	FokI
Ingles [47]	2001	United States	Caucasian	373/394	62.3	62.2	PCR-RFLP	Yes	FokI
Kim [48]	2001	United States	Caucasian	393/406	57.9 ± 9.7	53.0 ± 10.9	Taqman	Yes	BsmI
Slattery [49]	2001	United States	Caucasian	424/266	NA	NA	PCR-RFLP	Yes	FokI, BsmI, TaqI

VDR, vitamin D receptor; PCR, polymerase chain reaction; PCR-RFLP, polymerase chain reaction-restriction fragment length polymorphism; ASM-PCR, allele specific multiple-PCR; HWE, Hardy–Weinberg equilibrium; NA, **Not available**.

### Heterogeneity and publication bias

The heterogeneity was assessed for each study using the *Q* statistic. Significant heterogeneity (*P* for heterogeneity < 0.10 or *I^2^* > 50%) between studies were observed in *BsmI* and *ApaI*, but no heterogeneity was found in *FokI*, *TaqI* and *Cdx2* polymorphisms.

Funnel plot and Egger’s test were performed to evaluate the publication bias of literatures on CRC, and no statistically significant publication biases were found in all genetic models.

### Meta-analysis results

#### FokI polymorphism and CRC

A total of 29 studies examined the association between CRC and the *FokI* polymorphism. The result of meta-analysis showed that the *FokI* polymorphism was on the fringe of statistically significant in the comparison of F allele vs. f allele in fixed model (OR = 1.029, *95%CI* = 0.999–1.059, *P*^raw^ = 0.057, *P_FDR_ =* 0.057). The homozygous model, the dominant model and the recessive model were no significant associated with CRC risk (Table 2).

**Table 2:** Meta-analysis of the association between VDR polymorphisms and CRC

SNP	Comparison	Qualified studies	OR (95%CI)	*P*-value	FDR	Heterogeneity test	Effect model
FokI	F vs. f	29	1.029 (0.999–1.059)	0.057	0.057	*P* = 0.003, I*^2^* = 46.8%	F
	FF vs. ff		1.055 (0.990–1.123)	0.097	0.211	*P* = 0.015, I*^2^* = 39.8%	F
	FF + Ff vs. ff		1.045 (0.986–1.107)	0.141	0.211	*P* = 0.124, I*^2^* = 23.9%	F
	Ff + ff vs. FF		0.974 (0.876–1.083)	0.625	0.625	*P* < 0.001, I*^2^* = 81.5%	R
BsmI	B vs. b	23	0.862 (0.761–0.976)	0.019^*^	0.019^*^	*P* < 0.001, I*^2^* = 91.4%	R
	BB vs.bb		0.786 (0.636–0.972)	0.026^*^	0.039^*^	*P* < 0.001, I*^2^* = 85.5%	R
	BB + Bb vs. bb		0.824 (0.705–0.964)	0.015^*^	0.039^*^	*P* < 0.001, I*^2^* = 88.0%	R
	Bb + bb vs. BB		0.887 (0.759–1.036)	0.129	0.129	*P* < 0.001, I*^2^* = 78.8%	R
ApaI	A vs. a	12	1.025 (0.928–1.132)	0.631	0.631	*P* < 0.001, I*^2^* = 68.9%	R
	AA vs. aa		0.953 (0.775–1.172)	0.650	0.900	*P* < 0.001, I*^2^* = 67.9%	R
	AA + Aa vs. aa		1.009 (0.875–1.163)	0.900	0.900	*P* = 0.003, I*^2^* = 59.8%	R
	Aa + aa vs. AA		0.901 (0.770–1.055)	0.197	0.591	*P* = 0.001, I*^2^* = 65.5%	R
TaqI	T vs. t	18	1.011 (0.960–1.066)	0.673	0.673	*P* = 0.081, I*^2^* = 33.1%	F
	TT vs. tt		1.027 (0.912–1.157)	0.656	0.746	*P* = 0.091, I*^2^* = 32.5%	F
	TT +Tt vs. tt		1.018 (0.913–1.136)	0.746	0.746	*P* = 0.069, I*^2^* = 35.4%	F
	Tt + tt vs. TT		1.013 (0.944–1.086)	0.724	0.746	*P* = 0.310, I*^2^* = 11.8%	F
Cdx2	C vs. c	4	0.936 (0.828–1.058)	0.287	0.287	*P* = 0.352, I*^2^* = 8.2%	F
	CC vs. cc		0.862 (0.627–1.186)	0.363	0.544	*P* = 0.193, I*^2^* = 36.6%	F
	CC + Cc vs. cc		0.933 (0.723–1.204)	0.594	0.594	*P* = 0.176, I*^2^* = 39.3%	F
	Cc + cc vs. CC		0.918 (0.783–1.077)	0.293	0.544	*P* = 0.777, I*^2^* = 0.0%	F

VDR, vitamin D receptor; CRC, Colorectal cancer; OR, odds ratios; 95% CI, 95% confidence interval; FDR: *p* value from Benjamini–Hochberg method control for false discovery rate (FDR); R, random-effects model; F, fixed-effects model; ^*^statistical significance.

#### *BsmI* polymorphism and CRC

**Figure 2:**
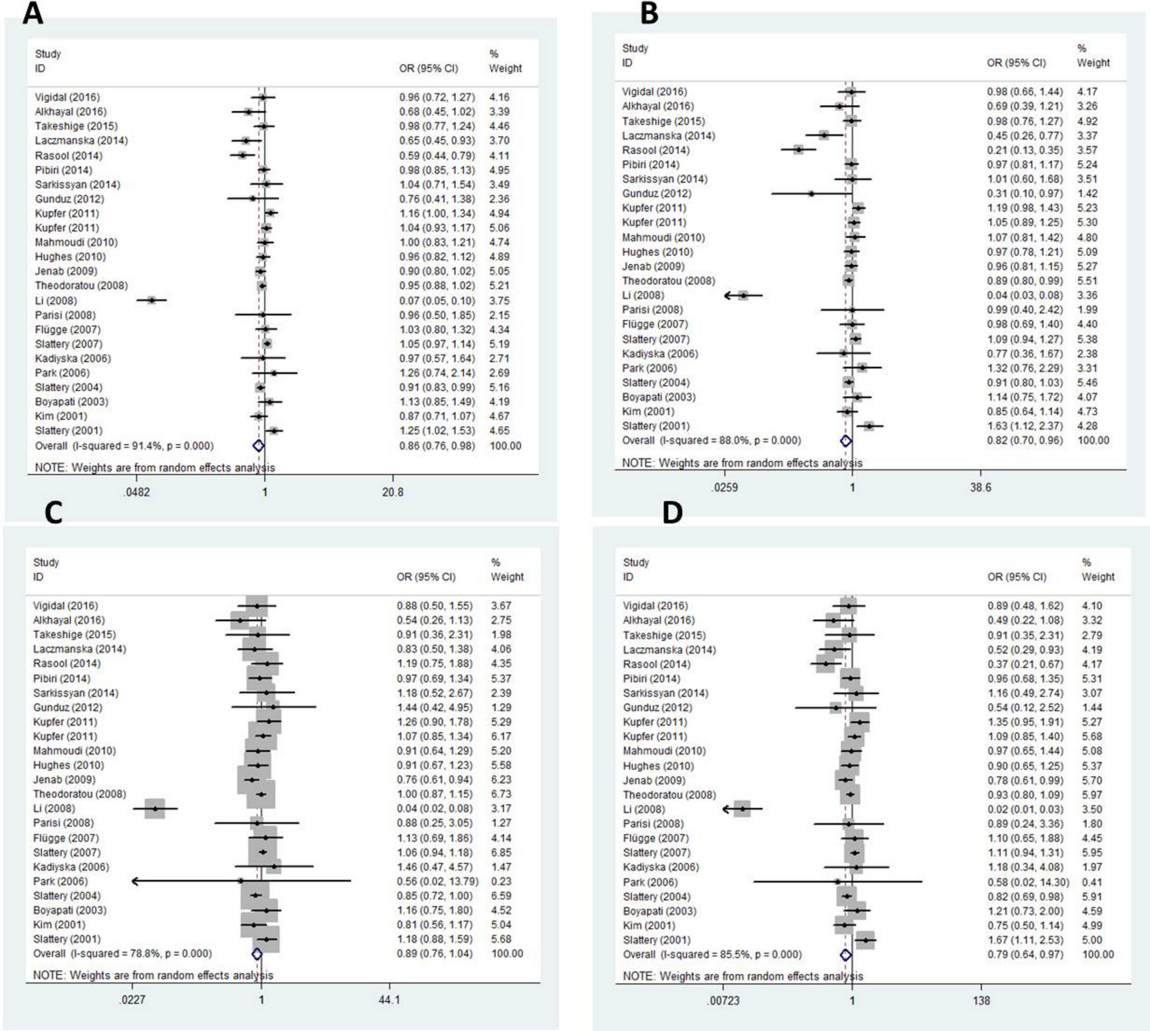
Forest plots for *BsmI* gene polymorphism and CRC (**A**) Allelic mode (**B**) Dominant mode (**C**) Recessive model (**D**) Homozygous model.

There were 23 articles on the relationship between *BsmI* polymorphism and CRC. We observed significant differences in allele frequencies, the homozygous model and the dominant model between CRC patients and healthy controls (B vs. b: OR = 0.862, *95% CI* = 0.761–0.976, *P*_raw_ = 0.019, *P*_FDR_*=* 0.019; BB vs. bb: OR = 0.786, *95% CI* = 0.636–0.972, *P*_raw_ = 0.026, P_*FDR*_ *=* 0.039; BB + Bb vs. bb: OR = 0.824, *95% CI* = 0.705–0.964, *P*_raw_ = 0.015, *P*_FDR_ *=* 0.039, respectively). There was little evidence of significant differences that investigated an association between *BsmI* polymorphism and CRC in the recessive model (Table 2, Figure 2).

### Other polymorphisms and CRC

Other three polymorphisms including *ApaI*, *TaqI*, *Cdx2* were not associated with CRC in all genetic models.

### Sensitivity analysis

Sensitivity analysis was performed by sequential omission of individual studies. The pooled ORs of the polymorphisms were not altered after omission, indicating that our results were statistically robust.

## DISCUSSION

The pathogenesis of CRC remains unknown. Gene-environment interactions, gene-gene interactions and life-style have an important impact on the development of CRC. There is consistent epidemiologic evidence that increased vitamin D intake is associated with reduced risk of colorectal. *VDR* mediate the biological activity of vitamin D and plays a crucial role in the etiology and development of cancer. A number of genetic associations studies were carried out to investigate the association of *VDR* polymorphisms with CRC risk, but the results are conflictive and the effect of *VDR* polymorphisms on CRC remains unclear. Therefore, in order to overcome the limitations of individual studies, we performed meta-analysis to evaluate the associations of *VDR* polymorphisms with CRC risk. Meta-analysis increases statistical power and resolution by pooling the results of independent analyses. A total of 52 reports had predicted a potential genetic association, and only 39 articles were included in this meta-analysis based on the selection criteria. The meta-analysis showed that the *FokI* polymorphism was on the fringe of statistically significant in the comparisons of F vs. f (OR = 1.029, *95% CI* = 0.999–1.059, *P_raw_* = 0.057, *P_FDR_ =* 0.057) and the *BsmI B* allele was associated with a lower CRC risk (B vs. b: OR = 0.862, *95% CI* = 0.761–0.976, *P_raw_* = 0.019, *P_FDR_* *=* 0.019). Similarly, a decreased CRC risk was also found in the homozygous model and the dominant model of *BsmI* (BB vs. bb: OR = 0.786, *95% CI* = 0.636–0.972, *P_raw_* = 0.026, *P_FDR_* *=* 0.039; BB + Bb vs. bb: OR = 0.824, *95% CI* = 0.705–0.964, *P_raw_* = 0.015, *P_FDR_* *=* 0.039, respectively). The results are consistent with the previous meta-analysis, which further confirmed the conclusions of the previous meta-analysis. However, our results were not consistent with the previous meta-analysis in the recessive model of *BsmI* and CRC. Yu et al. [3] and Bai et al. [51] draw the conclusion that the recessive model of *BsmI* was associated with a decreased CRC risk. The reasons for different results are as follows: first, our study is an updated and more carefully selected study than Yu et al and Bai et al. Second, our study included more Asian population. The estimated VDR polymorphisms including *FokI*, *ApaI*, *TaqI* and *Cdx2* showed no significant associations between CRC. Previous meta-analysis’s pooled ORs were similar to ours. In addition, we found significant heterogeneities between studies in *BsmI* and *ApaI*. But the reasons for the heterogeneity were unclear. The heterogeneity may be explained by the following factors: the study design, clinical characteristics, year of publication, and especially the different genetic backgrounds.

As in any study, some limitations of this study should be considered. First, only published studies in English and Chinese were included in this meta-analysis, so publication bias may have occurred. Second, significant heterogeneity was observed in overall comparisons. Although no publication bias was observed, different background and variant adjusted factors of controls were possible major source of heterogeneity. Third, although environment and diet may partially contribute to CRC, gene-gene and gene-environment interactions could not be investigated. Fourth, meta-analysis was still an observational study that subjected to the methodological deficiencies of the included studies.

In conclusion, this meta-analysis suggests that *BsmI* was associated with CRC risk and *FokI* might be risk factors for CRC. However, these associations with CRC need further studied.

## MATERIALS AND METHODS

### Literature search strategy

All genetic association studies that assessed the associations of the *FokI*, *BsmI*, *ApaI*, *TaqI* and *Cdx2* polymorphisms in the *VDR* genes with CRC susceptibility were included/enrolled in the meta-analysis. The studies were identified by extended computer based search of The PubMed, Web of Science, the Chinese Biomedical Database (CBM) and Chinese National Knowledge Infrastructure (CNKI) and Wanfang (Chinese) databases (published until April 2017). The keywords “Colorectal cancer” or “CRC” or “Colorectal carcinoma” or “Colorectal tumor”, “polymorphism” or “variant” or “genes” or “genotypes” or “genotyping”, “vitamin D receptor” or “*VDR*” were used. All references cited in the publications were also reviewed to identify other relevant publications. Finally, only published studies with full text were included.

### Inclusion and exclusion criteria

Regarding CRC susceptibility and *VDR* gene polymorphisms, studies which satisfy all the following criteria were identified: (1) articles investigate the associations of the *FokI*, *BsmI*, *ApaI*, *TaqI* and *Cdx2* polymorphisms in the *VDR* genes with the development of CRC; (2) a case–control study; (3) articles reported the number of individual genotypes and/or alleles for *VDR* polymorphisms in cases and controls; (4) the paper should clearly describe CRC diagnoses; (5) the control’ ethnic background and geographic area were the same with case’; (6) the language of articles was restricted to English or Chinese; (7) full text was available. Exclusion criteria: (1) the study was conducted on animals; (2) abstracts, case reports, editorials and review articles were excluded; (3) studies that did not met the inclusion criteria; (4) study with no detailed data.

### Data extraction

According to the selection criteria, data from relevant studies were carefully and independently extracted by two authors (Zhipeng Pan and Mengya Chen). Disagreement was resolved by discussion and consultation with the third researcher (Xingxing Hu). The following data were extracted if available: first author, year of publication, country, ethnicity of study population, the genotyping method, sample size, number of each genotype in cases and controls.

### Statistical analysis

The strength of the associations between the *VDR* polymorphisms and CRC susceptibility were evaluated by Odds ratios (ORs) with 95% confidence intervals (95% *CIs*) under the appropriate genetic model. The pooled ORs were calculated for the allele contrasts, homozygous model, recessive genetic model and dominant genetic model. *P* value < 0.05 was considered to be statistically significant comparing CRC cases with controls. Considering the possibility of heterogeneity in the studies, heterogeneity assumption was measured by the chi-square based *Q* test (*P* < 0.1 indicates heterogeneity) [52]. In addition, the presence of heterogeneity between studies was tested by the *I^2^*. *I^2^* values of 25, 50, and 75% are defined as low, moderate, and high estimates, respectively. The pooled effect was calculated by a fixed effect model when there is no heterogeneity (*I^2^* < 50% or *P* > 0.1), otherwise, a random effects model was used. HWE was assessed by the Chi-square test in the control group of each study in all the included studies (*P* < 0.05 was considered significant). The funnel plot and Egger’s regression test were used to search for publication bias, and an asymmetric Funnel plot or *P* < 0.05 in Egger weighted regression suggested possible publication bias. In consideration of multiple comparisons, Benjamini–Hochberg (BH) method was applied to control the false discovery rate (FDR). All the statistical manipulations were performed using the STATA statistical software 11.0 (StataCorp, College Station, TX, USA) and Review Manager Software 5.1 (Cochrane Collaboration, Oxford, UK). All *P* values tested were two-tailed.

## Abbreviations

CRC: Colorectal cancer
*VDR*: vitamin D receptor
CBM: the Chinese Biomedical Database
CNKI: Chinese National Knowledge Infrastructure
OR: odds ratio
95% *CIs*: 95% confidence intervals
FDR: false discovery rate
